# Penetration of foliar-applied Zn and its impact on apple plant nutrition status: in vivo evaluation by synchrotron-based X-ray fluorescence microscopy

**DOI:** 10.1038/s41438-020-00369-y

**Published:** 2020-09-01

**Authors:** Ruohan Xie, Jianqi Zhao, Lingli Lu, Patrick Brown, Jiansheng Guo, Shengke Tian

**Affiliations:** 1 MOE Key Laboratory of Environment Remediation and Ecological Health, College of Environmental and Resource Science, Zhejiang University, Hangzhou, 310058 China; 2 Zhejiang Provincial Key Laboratory of Subtropic Soil and Plant Nutrition, Zhejiang University, Hangzhou, 310058 China; 3 Department of Plant Sciences, University of California, Davis, CA 95616 USA; 4 Department of Pathology of Sir Run Run Shaw Hospital, Zhejiang University School of Medicine, Hangzhou, 310020 China; 5 Center of Cryo-Electron Microscopy, Zhejiang University School of Medicine, Hangzhou, 310058 China

**Keywords:** Plant physiology, Stomata

## Abstract

The absorption of foliar fertilizer is a complex process and is poorly understood. The ability to visualize and quantify the pathway that elements take following their application to leaf surfaces is critical for understanding the science and for practical applications of foliar fertilizers. By the use of synchrotron-based X-ray fluorescence to analyze the in vivo localization of elements, our study aimed to investigate the penetration of foliar-applied Zn absorbed by apple (*Malus domestica* Borkh.) leaves with different physiological surface properties, as well as the possible interactions between foliar Zn level and the mineral nutrient status of treated leaves. The results indicate that the absorption of foliar-applied Zn was largely dependent on plant leaf surface characteristics. High-resolution elemental maps revealed that the high binding capacity of the cell wall for Zn contributed to the observed limitation of Zn penetration across epidermal cells. Trichome density and stomatal aperture had opposite effects on Zn fertilizer penetration: a relatively high density of trichomes increased the hydrophobicity of leaves, whereas the presence of stomata facilitated foliar Zn penetration. Low levels of Zn promoted the accumulation of other mineral elements in treated leaves, and the complexation of Zn with phytic acid potentially occurred owing to exposure to high-Zn conditions. The present study provides direct visual evidence for the Zn penetration process across the leaf surface, which is important for the development of strategies for Zn biofortification in crop species.

## Introduction

Zinc (Zn) is a vital micronutrient and is involved in various cellular processes in living organisms. In addition, Zn has a critical function in maintaining the structure of several types of proteins, such as transcription factors^1–3^. Zn deficiency in the soil can affect plant health, leading to poor yields. This can in turn affect the livelihood of people who depend on the harvest and can directly affect the health of individuals who feed on the harvested material^4,5^. This problem is much more severe with respect to the cultivation of fruit crop species because most fruit trees are severely affected by Zn deficiency^6,7^. Therefore, an interruption or decline in Zn supply substantially affects plant vegetative development and reproductive success in the subsequent year.

Zinc fertilization is a standard agricultural practice used to improve the Zn nutrition status of fruit trees. Nevertheless, correcting for Zn deficiency by soil supplementation can be a challenge, particularly in alkaline soils that are characterized by strong Zn fixation capacity. Moreover, the process usually requires the application of large quantities of fertilizer, which can be costly. In addition, because a large portion of fruit tree root systems penetrate deep soil layers, soil Zn treatments are frequently ineffective due to the poor mobility of Zn in soils^6^. Therefore, the use of foliar fertilizers is particularly useful for soils that limit Zn uptake by roots.

As opposed to the application of fertilizer in the soil, foliar application of nutrients guarantees fast and targeted uptake; that is, the nutrients can be delivered directly to the plant tissues during vital growth stages and during reproduction^8,9^, for example, during fruit development. However, foliar fertilizers are generally not designed on the basis of physiology and hence potentially suffer from various limitations. Foliar nutrient penetration is a complex process that relies on the leaf surface characteristics of plants, physicochemical properties of the chemicals, supplement type and concentration, and environmental conditions^9^. The plant leaf surface harbors various and complex collections of unique and specialized physical and chemical adaptations that shield plants against adverse environmental conditions and control gas, water, and nutrient exchange^10,11^. Many reports have shown that Zn foliar application to fruit trees can considerably boost Zn concentrations in the treated leaves; however, symptoms of Zn deficiency have been shown to remain in the untreated leaves, as well as in the leaves that form after the application^12–15^. The very limited retranslocation of Zn observed after foliar application may occur because of the limit of Zn penetration through the leaf surface or the tendency of leaf tissues to bind strongly to Zn. This limited understanding of the physiological processes of foliar-applied Zn absorption hinders our efforts to increase foliar Zn fertilizer effectiveness^8^.

Common problems when examining foliar penetration mechanisms include technical difficulties that are related to fluorescence and optical microscopy and trying to detect the absorption of specific solutes and dyes by micro- and nanostructures on the leaf surface^16^. To fundamentally gain insight into the effects of leaf surface properties on foliar Zn absorption, it is essential to go beyond the quantification of the total metal content in a bulk sample. Therefore, in our study, the in vivo Zn localization after foliar fertilizer treatment was investigated with the use of synchrotron-based X-ray fluorescence (XRF). This method can be used to investigate the distribution patterns of microelements applied via foliar sprays in plants with high sensitivity. This method could simplify foliar fertilizer development and application techniques that enhance nutrient translocation from application sites, which is among the key challenges affecting the foliar fertilizer industry^17^.

Apple (*Malus domestica* Borkh.) was selected as an experimental species because it is highly sensitive to Zn deficiency^18–20^. Most apple orchards in areas with calcareous and salt-affected soils suffer from Zn deficiency every year, which results in a high loss of yields and severe deterioration of fruit quality^18^. By using micro X-ray florescence, we were able to analyze the impact of the surface physicochemical properties of apple plant leaves on their Zn absorption and subsequent Zn translocation. High-resolution imaging was then applied to assess the penetration process by in vivo mapping of Zn at the subcellular level. We also investigated the interaction of Zn and other elements to examine how nutrition status in apple leaves responds to foliar Zn applications. The ability to visualize and quantify the pathway that elements take following application to leaf surfaces is critical for our understanding of the utility of foliar fertilizers and allows for the direct contrast of the relative efficacy of different formulations and chemistries.

## Materials and methods

### Plant cultivation

Apple plants were used in this study because of their high sensitivity to Zn deficiency and the heterogeneous topography between adaxial and abaxial leaf surfaces. Healthy, 5-year-old apple trees (*Malus domestica*) grafted onto *Malus hupehensis* (tea crabapple) rootstocks were grown in a greenhouse at Zhejiang University (latitude 36°28′N; longitude 120°15′E) in China. Each tree was planted in a 30-L container containing a mixture of soil:perlite (1:1) and watered with Hoagland nutrient solution as needed. The plants were maintained under natural light, as well as artificial light from lamps (16/8 h [day/night] photoperiod at 350–500 μmol m^−2^ s^−1^), at day/night temperatures of 20–26 °C and 50–70% relative humidity.

### Alterations of properties of plant leaf surfaces

To test the role of stomatal function on abaxial foliar Zn penetration, abscisic acid (ABA) and darkness treatments were used prior to foliar Zn application each time to control the stomatal aperture. Mature apple leaves were pretreated with foliar applications of 1 mM abscisic acid (ABA) and then maintained for 30 min in the dark to trigger stomatal closure, as previously described^21,22^. The effectiveness of the treatments was confirmed under light microscopy. To test the effect of trichome density on foliar Zn penetration, methyl jasmonate (MeJA) treatment and mechanical removal of trichomes were used to alter the lower epidermal structures. Abaxial trichomes on mature apple leaves were isolated mechanically by mildly scraping the surfaces of the leaves using a scalpel. Optical microscopy and scanning electron microscopy were used to monitor the removal effect. Methyl jasmonate (MeJA) was then applied to the new growth of young leaves as described previously^23,24^ until they were fully expanded. Growth lamps were switched off for 4 h following the application of MeJA. The effects of MeJA treatment on leaf properties were assessed by examining changes in cuticle thickness, cuticle composition, trichome density and stomatal density.

### Foliar application of Zn fertilizer

Fully expanded mature leaves of apple plants were selected and washed thoroughly with deionized water. A Zn solution (supplied as ZnSO_4_·7H_2_O, 200 ppm; pH 5.8) was applied onto one side of opposing leaves with a paintbrush. The concentration of Zn (200 ppm) was considered and selected on the basis of typical agricultural foliar Zn sprays. This application was repeated three times, with the full fertilization process lasting ~5 min. To avoid the influence of other factors on the leaves, no wetting agent or surfactant was added. The fertilizer was applied to the leaves 8 h before darkness, and the plants were covered using plastic bags, although the treated leaves were left uncovered to avoid unintentional spray application. Lanolin (Sigma-Aldrich Co., USA) and Teflon membranes were used to coat the leaf base petiole junction to protect the petioles of the treated leaves^25^. The Zn-based foliar application was repeated once daily. Parts of leaves were harvested after 3 d and then assessed by nano-XRF imaging; the other leaves were treated for 14 d and harvested 7 d later for micro-XRF imaging. Deionized water was used as a control. All the plant samples were gently rinsed by sequentially using 2% HNO_3_, 3% ethanol, and deionized water to remove the excess Zn immediately after harvest^23,26^. Each experiment included three replicates per treatment.

### Mineral element mapping by micro- and nano-XRF

Samples for micro-XRF analysis were cut at a thickness of 180 μm using a cryotome (Leica, CM1950, Leica Biosystems, Dahaner Corp., Germany) at −20 °C as previously described^20^. Micro-XRF imaging was conducted at the Stanford Synchrotron Radiation Laboratory (SLAC National Accelerator Laboratory, USA) with beam lines 2–3. The energy range of the BL2–3 optics covers 4.9–23 keV, with detection of fluorescent X-rays as low as those based on Si. A Kirkpatrick-Baez mirror system was used to achieve a microfocus with a beam size of ~2 × 2 microns. The flux was ~1 × 10^10^. The incident X-ray beam was microfocused using a pair of Kirkpatrick-Baez mirrors (Xradia Inc., Pleasanton, CA, USA) with the sample at 45° to the incident X-ray beam. The fluorescence yield was detected using a single-channel Vortex Si detector (Hitachi High-Technologies Science America Inc., CA, USA). Samples were attached onto sulfur-free tape and mounted on a standard sample holder. The experimental data were recorded at 13,500 eV using a 20-μm (H) × 20-μm (W) beam spot size and 100-ms dwell time pixel^−1^. Fluorescence signal intensities for these elements were determined using SMAK software. The integrated intensities of Zn were calculated from X-ray fluorescence spectra and normalized by the intensity of the Compton scattering peak. Elemental mapping for the measurement area was obtained from the normalized intensity for each element. The quantification of the fluorescence yield counts was normalized by that of I0 and the dwell time. The normalized X-ray fluorescence intensities were then scaled to different color brightness values for the individual elements, with the brightest spots corresponding to the highest elemental fluorescence. The scale of fluorescence counts for the individual elements was the same for each map. Samples for nano-XRF mapping were cut from different parts of the terminal bud and prepared using high-pressure freezing (HPF) followed by the freeze substitution (FS) method using acetone to preserve both the ultrastructure of samples as much as possible and the in vivo spatial distribution of elements during sample processing and analysis. Nano-XRF was performed in a helium atmosphere on an Advanced Photon Source 2-ID-D and 2-ID-E hard X-ray microprobe beamline (Argonne National Laboratory, USA)^24^. Incident X-rays of 10 keV were used to excite the tested elements. A Fresnel zone plate focused the X-ray beam to a spot size of 0.2 μm (H) × 0.2 μm (W) on the sample, with 20 ms pixel^−1^ dwell time. X-ray fluorescence of the sample was captured with an energy dispersive silicon drift detector. The resulting elemental maps were observed and analyzed using the MAPS program^27^.

### Speciation of zinc measured by K-edge XANES

Mature apple leaves treated with foliar-applied Zn were also collected for X-ray near-edge-structure (XANES) analysis. The plant samples were prepared according to our previous studies^28^. Six leaves collected from three independent plants were mixed and grown together under liquid nitrogen, and the powdered samples were pressed into 2-mm path length Lucite sample holders with Kapton tape windows cooled in liquid nitrogen. To minimize the breakdown and mixing of cellular components within the plant material, care was taken to keep the tissue frozen at all times during the measurements. During data collection, samples were maintained at ~10 K in a liquid helium flow cryostat to minimize the loss of intensity of the signal. The methods used to prepare standard Zn compounds were based on those of previous studies^20,29,30^. ZnSO_4_ was prepared as a 5 mM Zn solution in ultrapure deionized water. Zn_3_(PO_4_)_2_ was prepared as a 5 mM Zn solution by dilution with boron nitride. Complexes of Zn-malate, Zn-citrate, Zn-phytic acid, Zn-histidine, Zn-cysteine, and Zn-glutathione were prepared by adding 50 mM malate, citrate, phytate, histidine, cysteine, and glutathione to an aqueous solution of 5 mM Zn(SO)_4_. The pH of all the complex standards was adjusted to 6.0 using 0.5 M NaOH. Zn-nicotianamine was prepared by adding 20 mM nicotianamine to a 5 mM ZnSO_4_ solution, and the pH was adjusted to 7.0. Zn-polygalacturonic acid was prepared by adding 5 mM ZnSO_4_ to a 1% polygalacturonic acid solution. Thirty percent (v/v) glycerol was added to all the solutions, which were then mixed to minimize the formation of ice crystals during freezing. XANES data were collected at the SSRL with the storage ring SPEAR-3 operating at 3 GeV and with ring currents of 80–100 mA. X-ray absorption spectroscopy of bulk tissues was carried out on SSRL beamline 7–3. BL7–3 is equipped with a 30-element Ge solid-state detector in addition to ionization chambers and Lytle/PIPS detectors. A dedicated LHe cryostat allows for routine low-temperature measurements. The flux was ~1 × 10^12^, and the incident X-ray intensity was monitored using a krypton (Kr)-filled ionization chamber. The monochromator energy of each spectrum was calibrated using Zn foil between the second and third ionization chambers; its absorption edge was calibrated to an edge of 9659 eV. Zn Kα fluorescence was recorded using a 30-element germanium detector (Canberra Industries, Meriden, CT, USA) equipped with Soller slits and copper (Cu) filters. During data collection, the samples were maintained at ~10 K in a liquid helium flow cryostat to minimize the loss of intensity of the signal. Sixteen scans were collected and averaged for each sample to improve the signal:noise ratio. The normalization of the EXAFS spectra was carried out according to standard methods using the SIXpack program suite. The spectra were normalized to unit step height using a linear pre- and postedge background subtraction and then transformed to k-space based on an *E*_0_ equal to 9659 eV. The *k*-function was extracted from the raw data by subtracting the atomic background using a cubic spline consisting of seven knots set at equal distances fitted to *k*^3^-weighted data; the *k*^3^-weighted x(*k*)-functions were Fourier transformed across the 1.5–12 Å^−1^ range using a Kaiser-Bessel window, with a smoothing parameter of 4. The *k*^*3*^-weighted EXAFS spectra recorded from the plants were least-square fitted across a wave vector (*k*) range of 1.5–12 Å^−1^ using a combination of Zn compounds as standards. Best fits were derived by incrementally increasing the number of fitted components and minimizing the fitted residual. The range for the fit was varied as a function of data quality and to test contributions from minor components. Target transforms (TTs) were carried out using the SIXpack program (version 1.4)^31^, and XANES data analysis was carried out using the Athena program suite (version Demeter 0.9.26) according to standard methods (https://bruceravel.github.io/demeter/documents/Athena/index.html.).

### Scanning electron microscopy

The samples were fixed with 2.5% glutaraldehyde in phosphate buffer (0.1 M, pH 7.0) for more than 4 h, after which they were rinsed three times for 15 min in phosphate buffer (0.1 M, pH 7.0). The samples were postfixed in 1% OsO_4_ in phosphate buffer for 1–2 h and rinsed three times in phosphate buffer for 15 min. The samples were dehydrated by a graded series of ethanol (30, 50, 70, 80, 90, and 95%) for ~15 min at each concentration and then dehydrated twice in alcohol for 20 min at each step. The dehydrated samples were coated with gold-palladium in a Hitachi Model E-1010 ion sputter for 4–5 min and subsequently observed via a Hitachi Model SU-8010 scanning electron microscope.

### Statistical analysis

The quantification of fluorescence intensities was statistically analyzed using the SPSS software package (version 12.0) and visualized by Origin 2019 software. The variance of the data sets was assessed by Fisher’s least significant difference test (*p* < 0.05 indicates significant and *p* < 0.01 indicates very significant) for each set of corresponding data. The calculation and visualization of relevant data about the spatial correlation between the micro-XRF intensity of Zn versus P was performed via R (version 3.6.0).

## Results

### Surface structure of the adaxial and abaxial sides of apple leaves and its impact on foliar Zn absorption

**Fig. 1:**
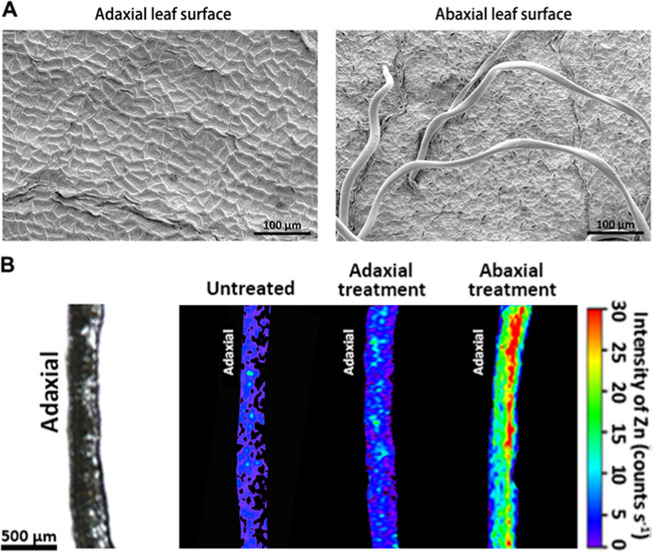
SEM micrographs of leaf surfaces of mature apple leaves and micro-XRF mapping of Zn in the cross‐sections of leaves after foliar Zn application. (**a**) SEM micrographs of leaf surfaces of apple mature leaves, showing that mature apple leaves have a heterogeneous topography between adaxial (left) and abaxial (right) leaf surfaces. (**b**) Micro-XRF mapping of Zn in the crosssections of leaves after foliar Zn application, showing the different efficiencies of Zn absorption in adaxial and abaxial leaf surfaces. In each section, the adaxial side is to the left. Fluorescence intensities (counts s-1) of Zn were normalized and scaled between red (maximum) and blue (minimum) for each map

To assess the overall leaf surface characteristics that affect the penetration barrier, the physiological properties of the adaxial and abaxial sides of apple leaves were determined (Fig. 1a). Surfaces of mature leaves of apple plants were observed using scanning electron microscopy (SEM), which revealed that mature apple leaves have a heterogeneous topography between their adaxial and abaxial surfaces, and the adaxial/abaxial trichome structure and morphology were similar to those described in previous studies of apple leaves. There was a smooth epicuticular wax layer covering the adaxial leaf surface of mature apple leaves; by contrast, the abaxial leaf side was covered by nonglandular trichomes. In addition, the adaxial side was completely astomatous (Fig. 1a).

To assess the differences in Zn absorption between the adaxial and abaxial leaf surfaces, micro-XRF was used to evaluated the leaf cross-sections after foliar Zn application. The X-ray fluorescence intensity was normalized and visualized with a heatmap that corresponded to differences in Zn concentration. Our results indicated a significant difference in the rate of Zn penetration between the adaxial and abaxial leaf surfaces (Fig. 1b). After continuous foliar Zn application for 2 weeks, very low signals for Zn were observed within the adaxial sides of treated leaves, while a greater distribution of Zn was observed in the abaxial sides of treated leaves.

### Comparison of Zn penetration processes between the surfaces of the adaxial and abaxial sides of leaves at the subcellular level

**Fig. 2:**
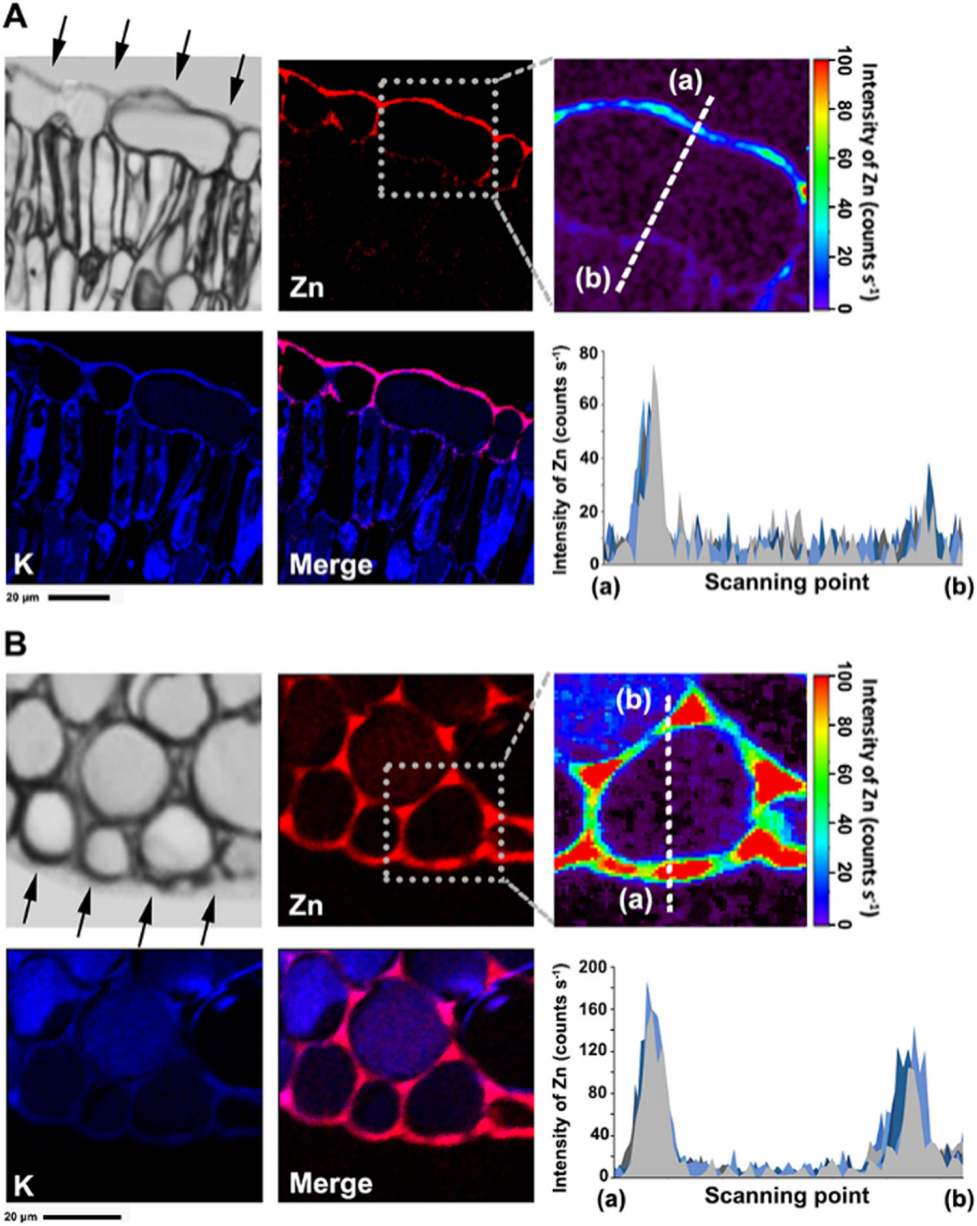
Subcellular distribution of Zn in apple leaves after foliar Zn application. Nano-XRF images were obtained from cross-sections of apple leaf tissue after foliar Zn treatment of adaxial and abaxial leaf surfaces. Subcellular distribution of Zn in apple leaves after foliar Zn application. Nano-XRF images were obtained from cross sections of apple leaf tissue after foliar Zn treatment of adaxial (**a**) and abaxial (**b**) leaf surfaces. The color merge image presents the relative locations of Zn (red) and K (blue). Pixel brightness is displayed in RGB, with the brightest spots corresponding to the highest content for the element depicted. Fluorescence intensity values (counts s-1) of Zn were normalized and 10 different scanning lines were selected trough the epidermal cells from point (**a**) to (**b**)

We conducted high-resolution imaging (nano-XRF) to further investigate the in vivo Zn distribution within the subcellular compartments of apple leaves after foliar Zn treatment. Foliar Zn penetration of the adaxial and abaxial leaf surfaces was visualized with merged images that showed the relative locations of Zn (red) and K (blue) (Fig. 2a, b). Treatment with Zn fertilizer for 3 d led to a significant increase in Zn levels detected around the upper and lower epidermal cells, with deposition in a narrow band that corresponded to the cuticle layer and epidermal cell walls. The Zn intensities (counts s^−1^) were also determined along the scan lines that passed through the epidermal cells. It is noteworthy that the detected Zn signals peaked in the cell walls and decreased sharply in the palisade parenchyma (Fig. 2a). This suggested that Zn was abundantly sequestered within the cell walls after penetrating the surface barriers. The Zn signals in the lower epidermal cell walls were ~3-fold higher than those in the upper epidermal cell walls.

### Effects of leaf surface properties on foliar Zn absorption

**Fig. 3:**
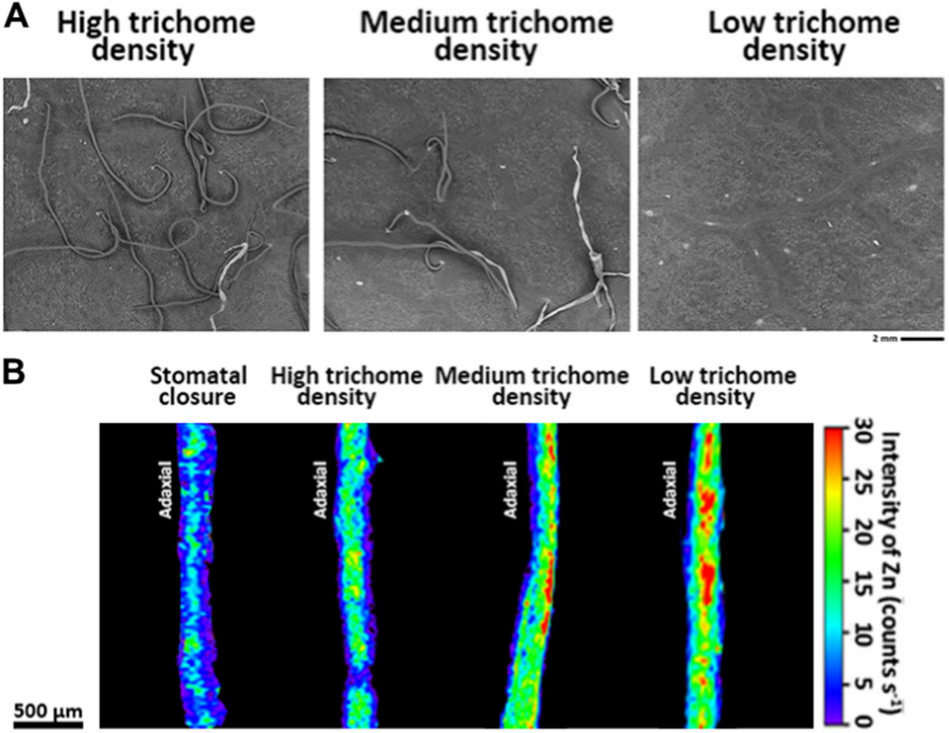
SEM micrographs of abaxial apple leaves with high, medium, and low trichome densities and micro-XRF mapping of Zn in cross‐sections of leaves with different physiological surface properties after foliar Zn application. (**a**) SEM micrographs of abaxial apple leaves with high, medium and low trichome densities. (**b**) Micro-XRF mapping of Zn in crosssections of leaves with different physiological surface properties after foliar Zn application, which show that the stomatal aperture and trichome density affect foliar-Zn uptake. Pretreatment with methyl jasmonate (MeJA) (A, left panel) and mechanical trichomes removal (A, right panel) were conducted to alter trichome density. Un-pretreated apple mature leaves were considered as having medium trichome density (A, middle panel). Fluorescence intensities (counts s-1) of Zn were normalized and scaled between red (maximum) and blue (minimum) for each map

We further evaluated the physiological properties of leaf surfaces that influenced the adsorption and penetration of Zn after foliar application. We studied the effects of stomatal aperture and trichome density on Zn penetration at the abaxial leaf surface. Abscisic acid (ABA) and darkness treatment were used prior to foliar Zn application each time to control the stomatal aperture. Methyl jasmonate (MeJA) treatment and mechanical removal of trichomes were used to modify lower epidermal structures. MeJA is a phytohormone involved in plant defense systems, and it can alter the properties of plant leaf surfaces, such as trichome density. We began MeJA application at the start of new growth of young leaves until they had fully expanded. Furthermore, we examined the changes in cuticle thickness and composition, trichome density and stomatal density to assess the effects of MeJA on leaf properties. The SEM images showed that pretreatment with MeJA increased the trichome density of the abaxial leaf surface (Fig. 3a, left panel) compared with that of the controls (Fig. 3a, middle panel). Mechanical scraping of the trichomes completely removed the trichomes on the leaf surface (Fig. 3a, right panel). No differences were observed in the change in stomatal density between the MeJA and control treatments (Fig. S1). FTIR and TEM analyses were also used to investigate alterations in cuticle composition and thickness, but no significant differences were observed after MeJA treatment (Figs. S2-S3). Therefore, we focused on the role of trichome density in the process of foliar Zn penetration using apple leaves pretreated with MeJA and leaves with their trichomes removed.

The micro-XRF results clearly showed that foliar Zn application led to a substantial increase in Zn levels in underlying leaf tissues, indicating that foliar-applied Zn penetration and absorption through the leaf surface occurred in all the treatments, and treatment-dependent effects were observed (Fig. 3b). Pretreatment with ABA and darkness to induce stomatal closure resulted in a relatively low penetration of Zn compared with that in the other treatments, suggesting foliar-applied Zn passed into the leaf interior through open stomata. Foliar Zn absorption was also related to trichome density; specifically, the penetration of Zn fertilizer increased as the trichome density decreased. Compared with MeJA-treated leaves with relatively more trichomes, leaves that had their trichomes removed had more Zn across the leaf surface and retention within the treated leaves, revealing a significant reduction in foliar Zn penetration. The in vivo scanning maps for Ca, Mn, Fe, P, and S in the cross-sections of leaves with different physiological surface properties after foliar Zn application are shown in Fig. S4.

### Nutrition status of apple leaves in response to foliar Zn application

**Fig. 4:**
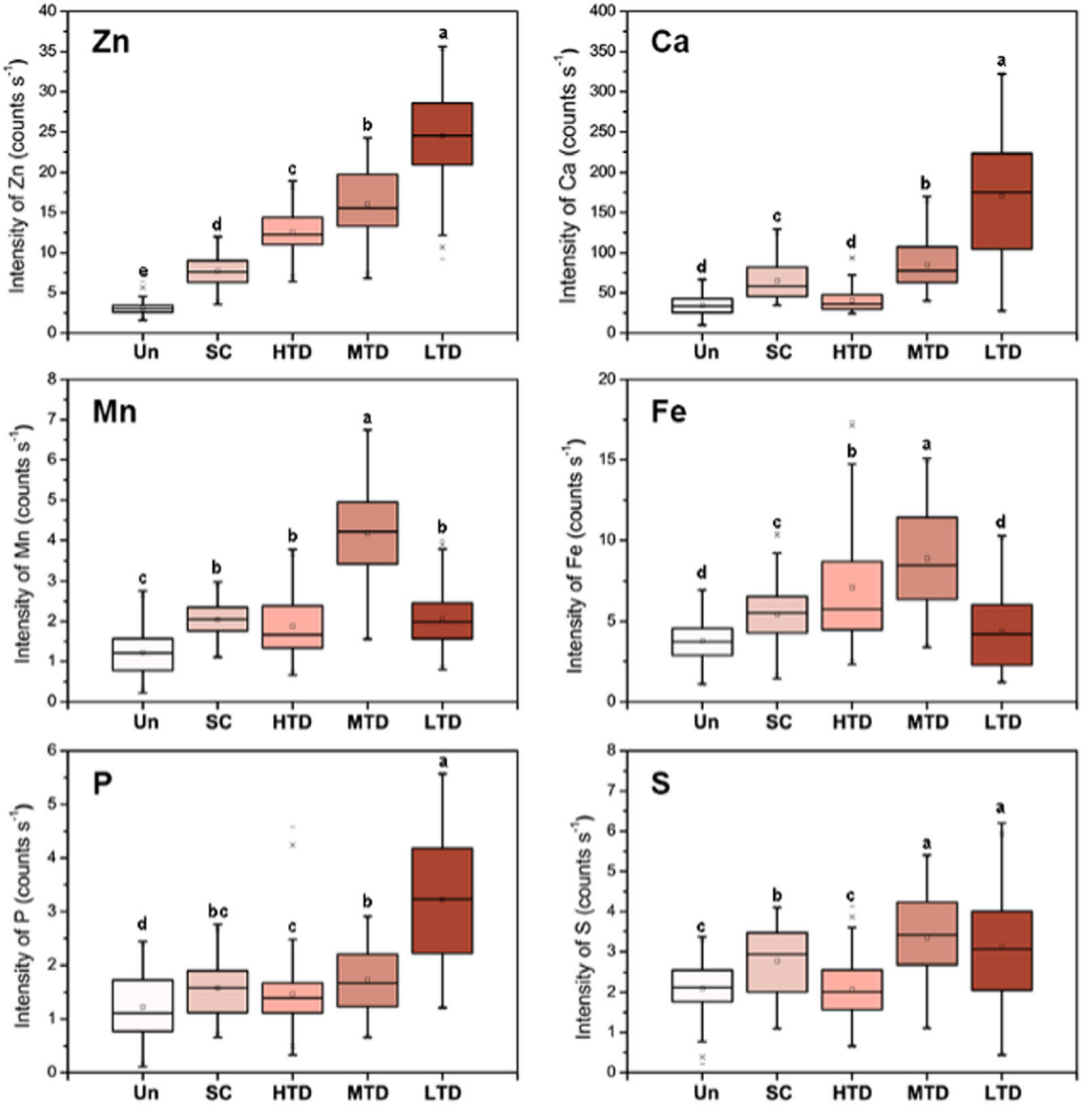
Fluorescence intensities (counts s^−1^) of Zn and other elements (Ca, Mn, Fe, P, and S) in the micro-XRF maps of leaves. Fluorescence intensities (counts s^-1^) of Zn and other elements (Ca, Mn, Fe, P and S) in the micro-XRF mapping of leaves. The lines at the top, bottom and middle of the box correspond to the 75th, 25th and 50th percentiles (median), respectively. Whiskers indicated ± standard deviation. The dots indicated the mean values. The variance was performed on the data sets by Fisher’s least significant difference test (*p* < 0.05 for significant and *p* < 0.01 for very significant) for each set of corresponding data. Un, untreated; SC, stomatal closure; HTD, high trichome density; MTD, medium trichome density; LTD, low trichome densities

The variation in elemental intensities and the correlations between different elements were further examined to reveal the possible impact of foliar Zn application on the nutrition status of treated leaves. Although it appears that the distribution patterns of other mineral elements (Ca, Fe, Mn, P, and S) shown in Fig. S4 were not obviously different between the treatments, the quantitation of their fluorescence yields helped to highlight the variation of individual elements after foliar Zn application. As shown in Fig. 4, the elements responded differently along with the increased Zn signals in the leaves. There is an induced effect on the tested elements after foliar application of Zn; among them, significantly induced enrichment of Ca and P was observed in leaves with low trichome density (LTD), in which Zn reached its highest levels. The concentrations of Mn and Fe changed depending on the Zn content, and low levels of Zn within leaves slightly promoted the accumulation of Mn and Fe, whereas high Zn concentrations in leaves had the opposite effect. The foliar application of Zn had no significant effect on the S concentration in the treated leaves (Fig. 4).

**Fig. 5:**
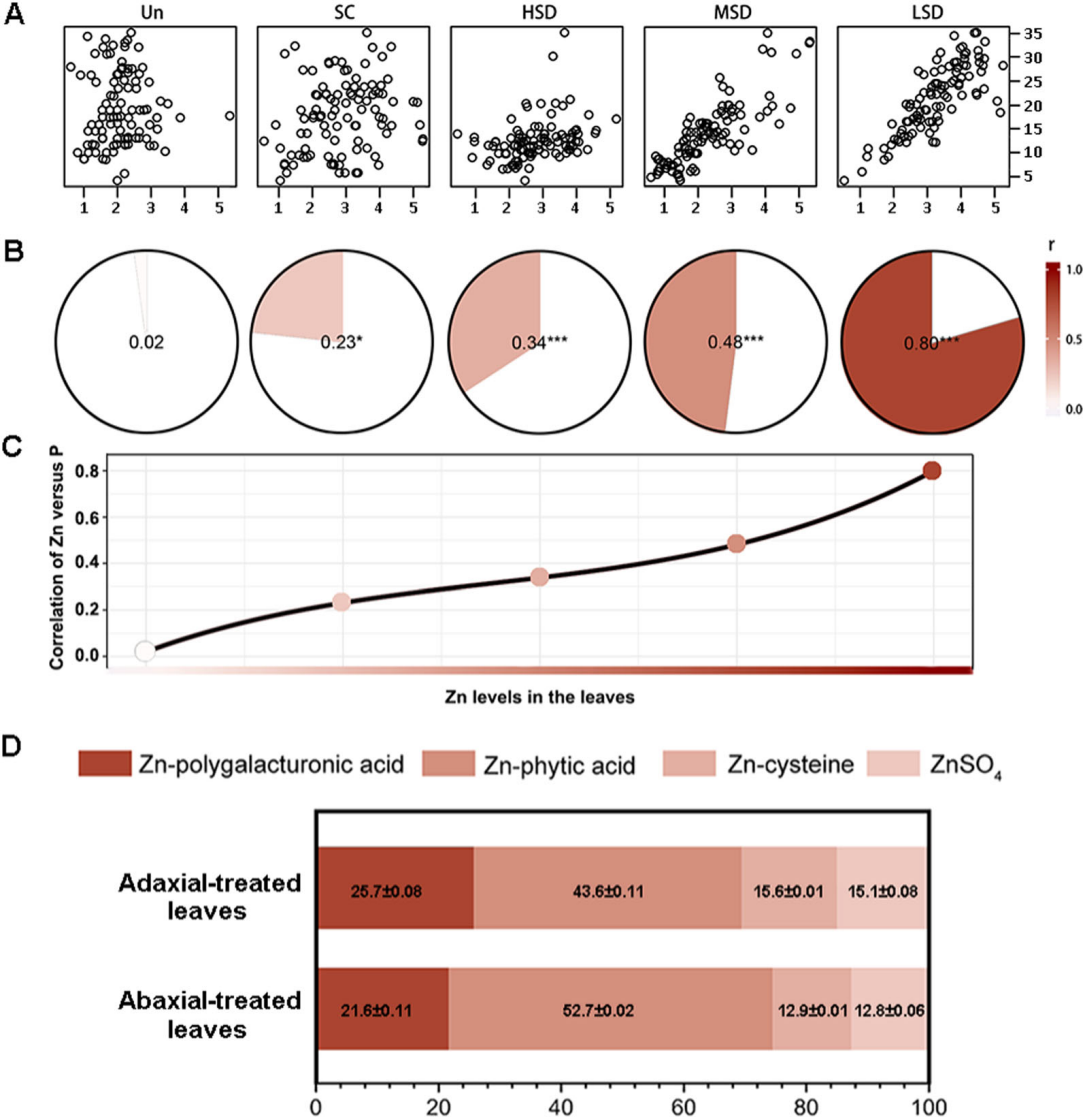
Spatial correlation of the micro-XRF intensity of Zn versus P and the proportion of Zn species in leaves. The spatial correlation between micro-XRF intensity of Zn versus P and the proportion of Zn species in leaves. (**a**) Scatter plot between intensity of Zn (Y-axis) and P (X-axis). (**b**–**c**) Trends of correlation coefficient (r) between intensity of Zn versus P, showing an enhanced spatial co-localization of Zn versus P in response to the increasing levels of Zn in the leaves after foliar Zn application. Asterisks indicate significant correlations by the Pearson test (***P < 0.001, *P < 0.05). (**d**) The proportion (% fraction) of Zn species in the apple leaf tissue after foliar Zn treatment of adaxial and abaxial leaf surfaces. Zn ligands were identified by linear combination fit (LCF) analyses of sample spectra using a combination of Zn model compounds as standard. Zn Kedge XANES recorded for Zn model compounds and samples, and the corresponding linear combination fits were shown in Fig. S5

The spatial correlations between Zn and other elements were further examined. There were no significant correlations between Zn and the tested elements (except P), in the distribution patterns. The Pearson correlation coefficients and their associated R values between Zn and P are presented in Fig. 5a–c. Our results revealed that there was an increased positive spatial correlation of Zn and P along with the penetration of foliar Zn fertilizer, suggesting a spatial overlap of Zn and P within the foliar treated leaves, and Zn was probably combined with P when the leaves were exposed to high exogenous Zn levels. We therefore performed XANES) analysis on powdered frozen-hydrated samples to acquire overall information about the speciation of Zn in leaves. By the use of linear-combination fitting (LCF), Zn was found to be present as Zn-polygalacturonic acid, Zn-phytic acid, Zn-cysteine, and ZnSO_4_ after foliar Zn application (Fig. 5d). It was predicted that the majority of Zn was coordinated by Zn-phytic acid, with 43.6 and 52.7% for leaves after foliar Zn treatment of adaxial and abaxial leaf surfaces, respectively. This demonstrated that phytic acid may play a role in the complexation and stabilization of foliar-absorbed Zn. Zn-polygalacturonic acid was the second most abundant Zn species. Polygalacturonic acid is a major component of pectin, which is most abundant in plant primary cell walls^32^. The XANES results confirmed the sequestration of Zn within cell walls during the penetration process across the leaf surface.

## Discussion

Currently, there is little information on the foliar penetration pathways of ionic solutes. The actual contribution of leaf surface structures to foliar-applied fertilizer absorption remains unclear^8^. A common problem of exploring the mechanisms of foliar penetration is the technical limitations related to fluorescence and optical microscopy and observing the penetration process of nutrients through the leaf surface^16^. In the present study, the direct visualization and high spatial resolution of XRF was suggested to be a promising and powerful strategy to investigate the distribution pattern of Zn within the plants following its application. By utilizing this technique, we were able to investigate the process of penetration of foliar-applied Zn across apple plant leaves with different physiological surface properties as well as help shed light on the possible interactions between foliar Zn concentration and the mineral nutrition status of treated leaves.

Our results showed a much higher efficiency in Zn penetration by abaxial surfaces than adaxial surfaces of mature apple leaves (Figs. 1 and 2). The greater nutrient absorption by the abaxial leaf surface may have resulted from the different physiological properties of the two leaf sides, as drop adherence to the leaf surface is a precondition for foliar penetration to occur^8,11^. The high degree of physical variation between the abaxial and adaxial leaf surfaces of apple leaves contributed to the interactions between the liquid fertilizer and the different leaf surfaces. The adaxial surface of apple leaves is covered with hydrophobic epicuticular wax and does not have stomata, which limits the bidirectional exchange of water, solutes and gases between the plant and the environment. Based on micro-XRF imaging, Du et al. also reported that foliar penetration of Zn was much more difficult through waxy leaves of citrus, though the concentrations of Zn increased significantly in the leaf tissues directly beneath the applied sites^26^. Very restricted nutrient solution absorption across the adaxial leaf surface also occurs in other fruit species, such as pear, lychee, and grapevine^32,33^. The extent of Zn mobilization following absorption also influences the efficacy of Zn penetration. In previous research, XRF was also performed to compare the behavior of different forms of foliar-applied Zn fertilizers, and the results indicated that Zn fertilizer applied as ZnSO_4_ was slightly more mobile than were other Zn forms, such as Zn-EDTA and ZnO^30,34^.

Further, we attempt to observe the distribution patterns of Zn at the subcellular level by using nano-XRF imaging with sufficient resolution and sensitivity. For this technique, sample preparation is very important. Here, a preparation procedure involving high-pressure freezing (HPF) followed by freeze substitution (FS) was used. This preparation protocol was proven in previous studies to preserve the ultrastructure of samples as much as possible, which can better preserve the in vivo localization of mobile elements^35–37^. This phenomenon was also confirmed in the present study, as the highly diffusible element, K (blue), was uniformly distributed in the palisade tissue and spongy tissue, which indicates that redistribution of the element did not significantly occur during sample preparation.

Recently, the cuticle was considered a lipidized epidermal cell wall region^38,39^. In the present study, the subcellular distribution of Zn occurred within a narrow band that corresponded to the cuticle layer and epidermal cells, which is consistent with the interpretation that the cuticle is the extension of the cell wall. In addition, this result provided direct visual evidence that there was a strong capability for Zn fixation in the cell wall following Zn application, which limited foliar Zn penetration and led to the low effectiveness of foliar Zn penetration. This was similar to the results of previous studies showing that Zn^2+^ has limited mobility in wheat leaves regardless of the form in which it is applied, which suggests a poor leaf penetration and high binding capacity of Zn to leaf tissues^30^. In contrast, other studies have shown that Zn moves across the cuticle of soybean and tomato leaves without binding to epidermal cells^23,40^, which may be due to the different plant materials used in the experiments and resulted in different interactions between the Zn solution and leaf surfaces. The cuticles of tomato and soybean leaves are much thinner, and therefore foliar absorption will be more rapid. Here, our findings indicate that a relatively high percentage of total Zn in the leaves from foliar applications would be bound to the cell wall rather than be present in a soluble, intercellular form. Zn mainly exists in a bound form in the cytoplasm, as well as in other cellular compartments, to avoid uncontrolled Zn^2+^ binding to nontarget sites^41^. Various studies have also shown that the abundance of negatively charged sites in the cell wall limits the translocation of positively charged Zn^2+ 8^. For example, the major component of pectin in cell walls is polygalacturonic acid, which has a high binding capacity for Zn^2+ ^^42^ Our previous research also shown that most of the Zn was deposited in the cell walls of apple leaves in coordination with different stages of development, with the nature of Zn binding being dependent on tissue age, as old and mature leaves exhibited higher proportions of Zn in the cell wall than young leaves^20^. In general, there is low potential for remobilization of foliar-absorbed nutrients until the potential binding sites for that element within the leaf are saturated. Therefore, we hypothesized that there would be very limited utilization of Zn applied to the adaxial leaf surface because of the highly hydrophobic cuticular wax layer that would limit Zn penetration, as well as having a higher capability for cationic Zn fixation.

Previous studies have suggested that there is high diversity among trichome structure and function, which can hinder or promote water penetration^11,43^. In the present study, we found that a relatively high trichome density resulted in a high degree of hydrophobicity (Fig. 3), which was not consistent with the results of several studies that reported that trichomes may participate actively in the absorption of water and foliar-applied nutrient solutions^44–46^. These differences may be related to the surface roughness provided by the high density, chemical composition, and structure of trichomes on apple leaves. The high density of nonglandular trichomes creates the hydrophobic characteristic of apple abaxial leaf surfaces to a certain degree, and repulsion of fertilizer drops by abaxial leaf trichomes could hinder the penetration of liquid Zn solutions. Our findings were similar to the results of Fernandez et al., who used Holm oak as a model to assess the capability of abaxial surfaces versus adaxial surfaces to absorb surface-deposited water drops^11^. Another study by Li et al. also demonstrated that trichomes are not part of the primary pathway through which foliar-applied Zn moves across the leaf surface, even though Zn was found to accumulate around the base of trichomes^23^. On the other hand, the possible contribution of stomata to the penetration of leaf-applied chemicals has been a matter of controversy for many decades, and it is still not fully understood whether stomata allow the penetration of foliar-applied solutes^16^. Nonetheless, several studies show a clear effect of stomata in promoting foliar solute penetration, even in the absence of surfactants^21,47^. However, the underlying mechanism is not understood, although it is hypothesized that penetration of solutes through stomata is restricted by their morphological and physical properties^8^. Our study provides further evidence that supports the relevance of stomatal nutrient absorption, as indicated by the increased penetration rates from plant surfaces whose stomata were present and open (Fig. 3).

Information about nutrient interactions will guide fertilization practices and optimize the efficiencies of fertilization strategies. The levels of two different elements for each pixel in an XRF data set can often provide important chemical information^48^. Here, we analyzed the ionic changes that occurred in leaves in response to foliar Zn application to reveal the interactions between and homeostasis of Zn and other metals. Induction of element accumulation was observed after foliar Zn penetration for all the tested nutrients (Fig. 4). One of the possible reasons for this promotional effect may be the important role of Zn in the constituents of enzymes involved in photosynthesis^49^. The concentration of water-soluble Zn in leaves was found to be closely correlated with the levels of chlorophyll. The positive influence of foliar Zn fertilizer on photosynthesis and chlorophyll synthesis may help increase mineral nutrient absorption and accumulation in functional mature leaves. A reduction in this accumulation was observed for Mn and Fe along with a further increase in Zn. This might be due to the possible Zn toxic reaction in the treated leaves because locally toxic levels of Zn may occur at the point of fertilizer application. Excess Zn may cause uncontrolled displacement of essential cofactor metal cations such as Mn^2+^ and Fe^2+ 3^. For example, it was reported that exposure to high Zn can subsequently inhibit PSII activity by replacing Mn in thylakoid membranes^50^. The underlying mechanisms of the above effects remain uncertain, and further research is needed to uncover the complex interaction between foliar Zn status and plant nutrient responses.

The increased spatial correlation of Zn versus P along with the penetration of foliar Zn fertilizer (Fig. 5a–c), together with the presence of Zn-phytic acid in the treated leaves (Fig. 5d), suggested that P may play an important role in the complexation of Zn, potentially in response to toxic concentrations of Zn. The formation of Zn-phytic acid in vegetative tissues was reported to occur under two circumstances: within some hyperaccumulator species or under conditions in which plants are exposed to high exogenous Zn concentrations, the binding of Zn to phytic acid helps plants limit Zn mobility and reduces toxicity^51–53^. Our results were consistent with the previous findings showing an increased proportion of Zn-phytate in wheat leaves after Zn-EDTA and ZnSO_4_ applications^30^.

Agronomic strategies aim to deliver phytoavailable Zn via the application of Zn fertilizers to leaves. The efficacy of foliar-applied Zn depends strongly on the successful absorption of the nutrient. Knowledge of the ability and mechanism of Zn to penetrate the leaf surface from the site of application and the factors that are associated with penetration efficiency is critical for the development of strategies for Zn biofortification of crops. In the present study, the high spatial resolution and direct imaging capability of XRF was valuable for distinguishing differences in Zn penetration of epidermal cells, and these techniques provided a powerful strategy for investigating foliar microelement applications to plants with a high level of sensitivity. By the use of micro- and nano-XRF techniques, our results provide direct visual evidence for Zn penetration of the leaf surface. Furthermore, we provide new insights that can help in the development of Zn biofortification approaches in fruit crops. To further increase the Zn content in food crops, future studies need to focus on elucidating the pathway by which Zn penetrates plant leaves, the subcellular compartmentation of Zn, and the specific formulation of Zn foliar fertilizers.

## Supplementary information

Supplementary figures S1-S5
