# Supplementary material for: Penetration of foliar-applied Zn and its impact on apple plant nutrition status: in vivo evaluation by synchrotron-based X-ray fluorescence microscopy
Source: Hortic Res. 2020 Sep 1;7:147. doi: 10.1038/s41438-020-00369-y (PMC7459125; doi:10.1038/s41438-020-00369-y)
Supplement: Supplementary file 1 — Supplementary figures S1-S5 [file 41438_2020_369_MOESM1_ESM.docx]

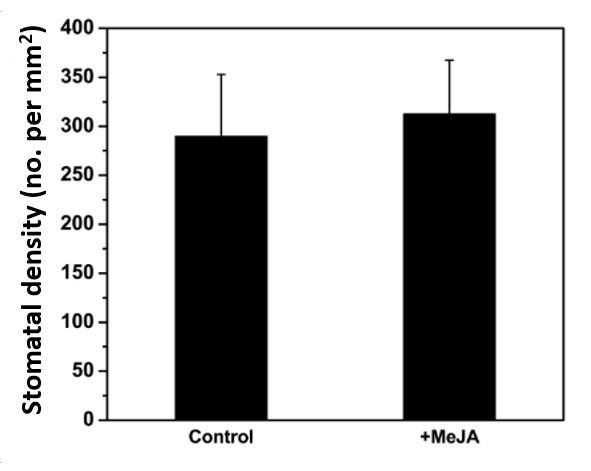


Fig. S1 Stomatal density on abaxial surface of mature apple leaves under MeJA and control treatments


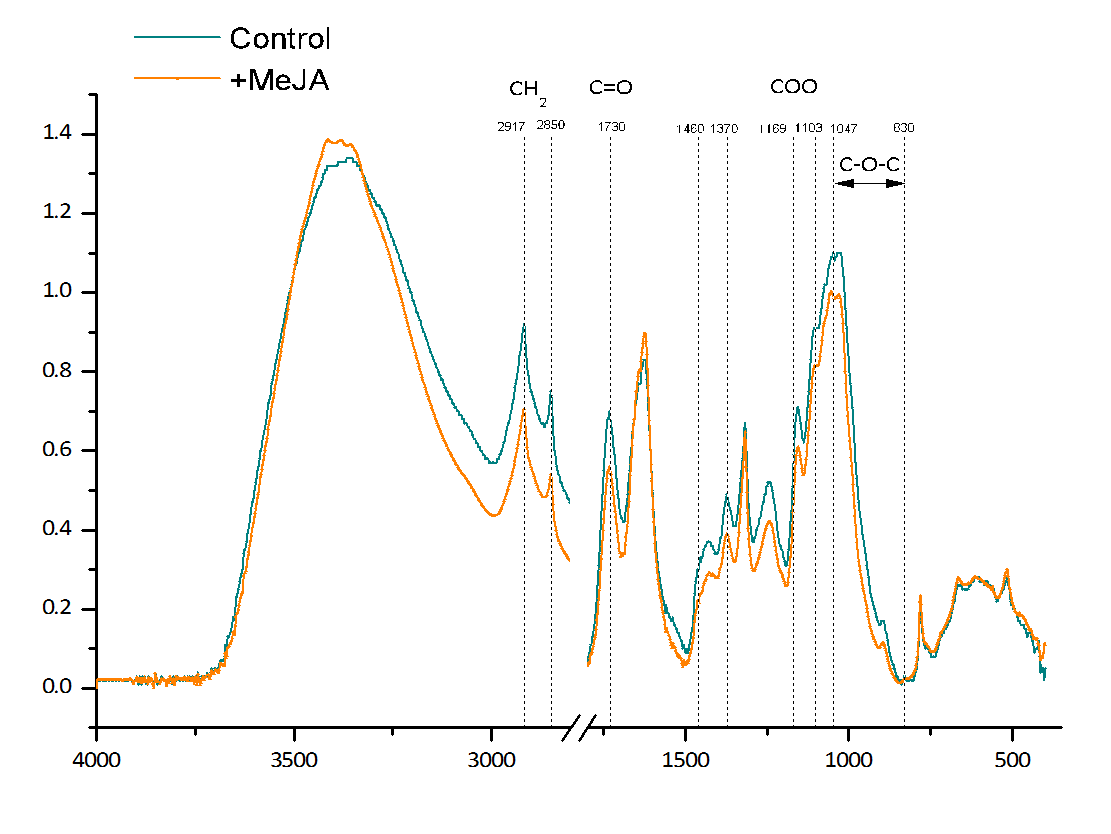


Fig. S2 Cuticle composition of leaves under MeJA and control treatments


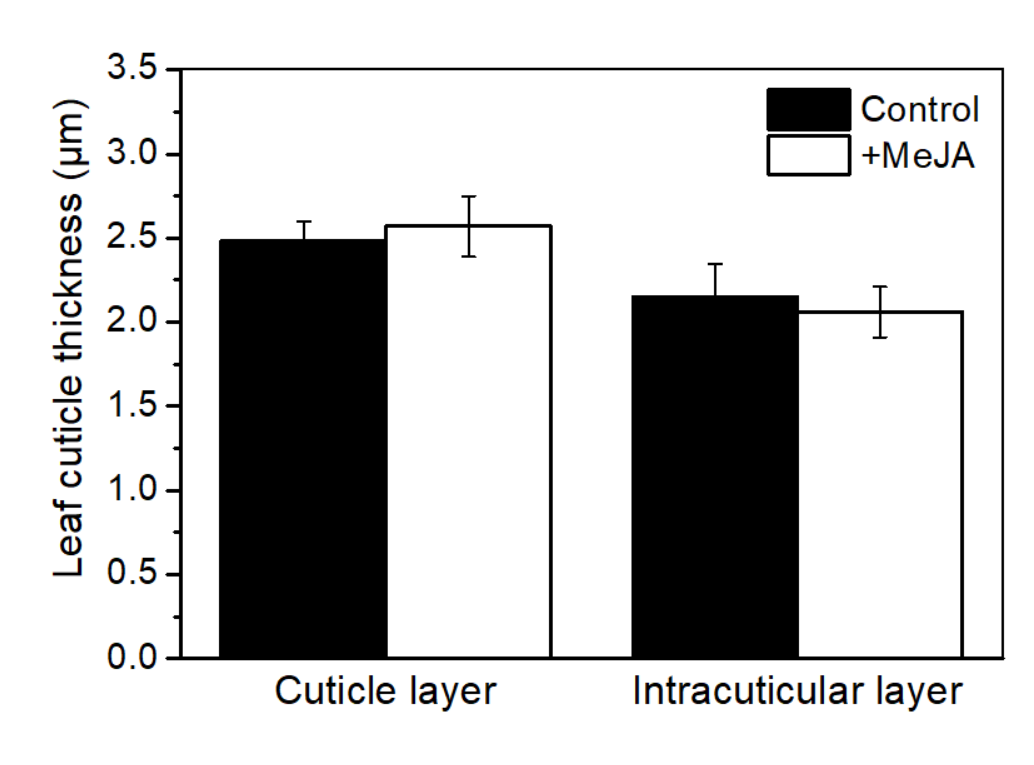


Fig. S3 Leaf cuticle thickness of abaxial surface from mature apple leaves under MeJA and control treatments

­­
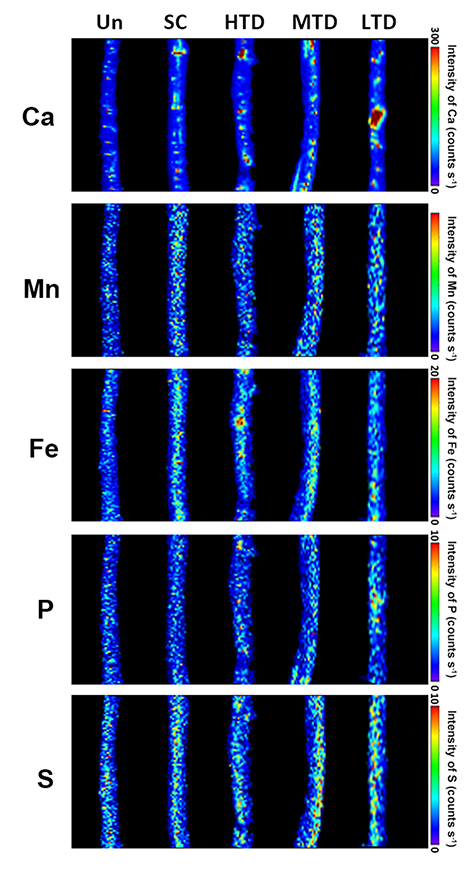


Fig. S4 Micro-XRF mapping of Ca, Mn, Fe, P and S in the cross‐sections of leaves after foliar Zn application. In each section, the adaxial side is to the left. Fluorescence intensities (counts s^-1^) of each element were normalized and scaled between red (maximum) and blue (minimum) for each map.


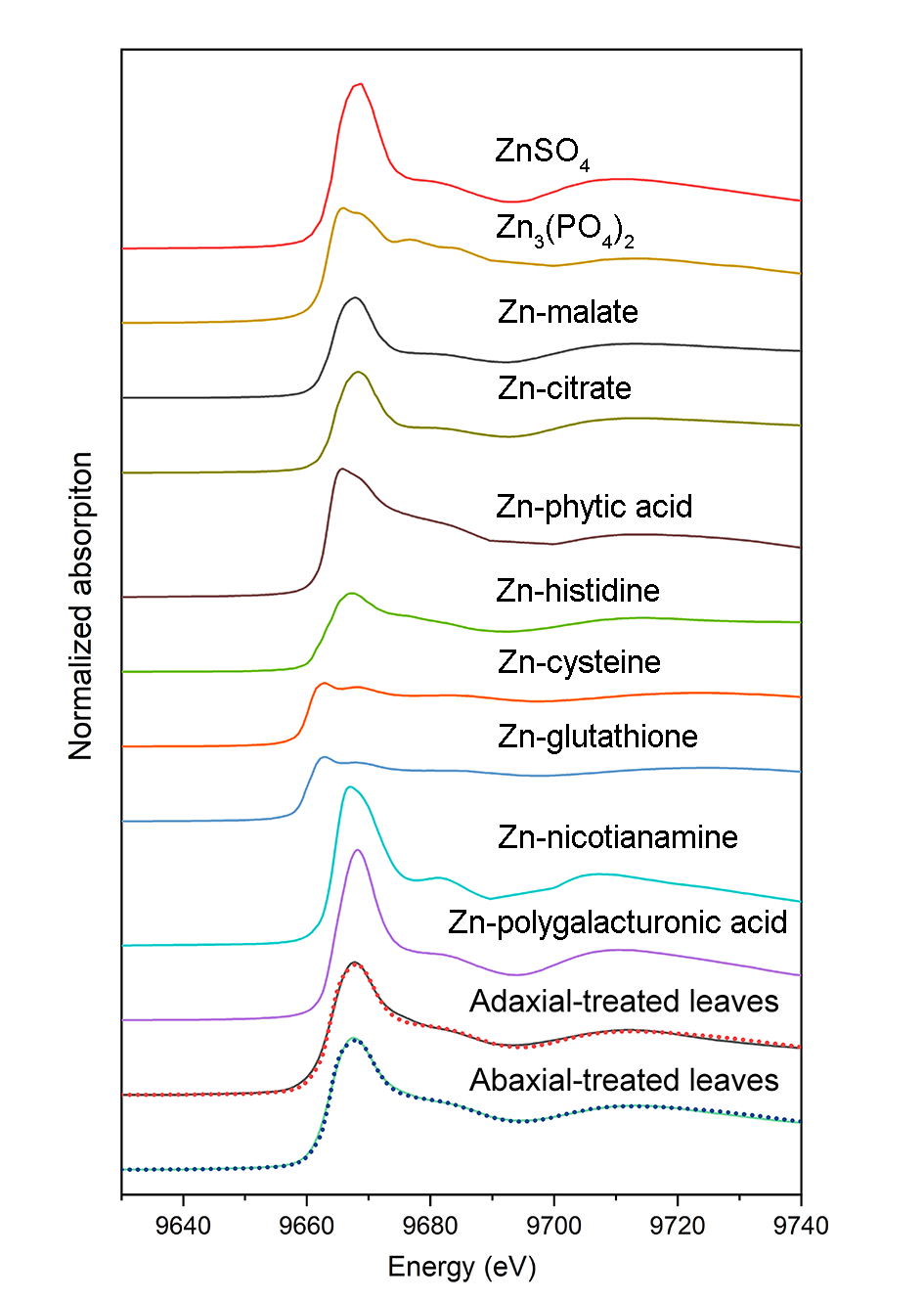


Fig. S5 Zinc (Zn) K‐edge XANES recorded for Zn model compounds and apple leaf tissue after foliar Zn treatment of adaxial and abaxial leaf surfaces (solid lines) with their linear combination fits (dashed lines)
